# Supplementary material for: The effects of video racing games on risk-taking in consideration of the game experience
Source: PLoS One. 2020 Oct 12;15(10):e0240367. doi: 10.1371/journal.pone.0240367 (PMC7549794; doi:10.1371/journal.pone.0240367)
Supplement: Attachment — Submitted filename: Response to Reviewers.docx [file pone.0240367.s002.docx]

**Response to Reviewers**

Dear Academic Editor and Reviewers,

Thank you very much for your feedback, comments and suggestions. The responses to your comments are as follows:

**Reviewer #1:**
*Comment 1: The Introduction Section is somewhat lengthy. It is suggested to describe the research background more concisely, or to move the part on previous studies into a Literature Review Section.* **\# The Introduction has been substantially shortened, and the research background is presented more concisely.**

*Comment 2: Some references on correlation test are suggested to add in the text, such as:
A multivariate random parameters Tobit model for analyzing highway crash rate by injury severity. Accident Analysis and Prevention, 2017, 99: 184-191.
Jointly modeling area-level crash rates by severity: A Bayesian multivariate random-parameters spatio-temporal Tobit regression. Transportmetrica A: Transport Science, 2019, 15(2): 1867-1884.
Spatial joint analysis for zonal daytime and nighttime crash frequencies using a Bayesian bivariate conditional autoregressive model. Journal of Transportation Safety and Security, 2020, 12(4): 566-585.*
**# These references have been added.**

*Comment 3: In addition, multicollinearity diagnoses can be also conducted (please refer to the above papers). Significant multicollinearity would have an adverse impact on the estimation results of mediation analysis.*
**# We attempted to investigate whether it is the game experience that underlies processes leading to increased risk-taking while playing video racing games. A mediation model is one of the best models to identify the mechanism that underlies an observed relationship between an independent and a dependent variable through the inclusion of a third hypothetical variable. This analysis is based on the multiple linear regression model. The multicollinearity analysis, in which one predictor variable in a multiple regression model can be linearly predicted from the others, is in this case not necessary to explain our hypothesis.**

*Comment 4: The practical implications of the analysis results should be pointed out. At end of the text, a Conclusion Section should be added to summarize the findings, to illustrate the limitations, and to provide some directions for future research.*
**# A Conclusion Section has been added.**

**Reviewer #2:**

*Comment 1: There are some typos in this paper. For example, "comprised30 men", "20minutes".*
**The typos have been corrected.**

*Comment 2: The reference style is not so correct. For example, "Fischer, Kubitzki (11)".*
**# The reference style has been edited.**

*Comment 3: Is there some information of the driving experience of the participants?*
**# Unfortunately, we have no information on the driving experience of the participants. It is important to note that the participants were randomly divided into two groups. The order of the games was also randomised in both groups. Therefore the statistical analysis has been performed appropriately.**

*Comment 4: For the correlation test, the author could refere to the following paper.
[1] Investigation on the Injury Severity of Drivers in Rear-End Collisions Between Cars Using a Random ParametersBivariate Ordered Probit Model, International Journal of Environmental Research and Public Health, 2019, 16(14), 2632.*
**# This reference has been added.**

*Comment 5: Some references related with driving simulation, maybe are helpful.
[2] Examining the safety of trucks under crosswind at bridge-tunnel section: A driving simulator study, Tunnelling and Underground Space Technology, 2019, 92, 103034.* [*https://doi.org/10.1016/j.tust.2019.103034*](https://doi.org/10.1016/j.tust.2019.103034) *[3] Examining the influence of decorated sidewaall in road tunnels using fMRI technology, Tunnelling and Underground Space Technology, Volume 99, 2020,* [*https://doi.org/10.1016/j.tust.2020.103362*](https://doi.org/10.1016/j.tust.2020.103362)
***#* These references have been added.**

Many thanks again.

Ewelina Stollberg and Klaus W. Lange
